# Bedside Ultrasound in Resuscitation and the Rapid Ultrasound in Shock Protocol

**DOI:** 10.1155/2012/503254

**Published:** 2012-10-24

**Authors:** Dina Seif, Phillips Perera, Thomas Mailhot, David Riley, Diku Mandavia

**Affiliations:** ^1^Department of Emergency Medicine, Los Angeles County+USC Medical Center, General Hospital, 1200 State Street, Room 1011, Los Angeles, CA 90033, USA; ^2^Division of Emergency Medicine, Stanford University Medical Center, 300 Pasteur Drive, Alway Building, M121, Stanford, CA 94305, USA; ^3^Division of Emergency Medicine, New York-Presbyterian Hospital, Columbia University Medical Center, 622 West 168th Street, New York, NY 10032, USA

## Abstract

Assessment of hemodynamic status in a shock state remains a challenging issue in Emergency Medicine and Critical Care. As the use of invasive hemodynamic monitoring declines, bedside-focused ultrasound has become a valuable tool in the evaluation and management of patients in shock. No longer a means to simply evaluate organ anatomy, ultrasound has expanded to become a rapid and noninvasive method for the assessment of patient physiology. Clinicians caring for critical patients should strongly consider integrating ultrasound into their resuscitation pathways.

## 1. Introduction

Early recognition and appropriate treatment of shock have been shown to decrease mortality [1, 2]. Incorporation of bedside ultrasound in patients with undifferentiated shock allows for rapid evaluation of reversible causes of shock and improves accurate diagnosis in undifferentiated hypotension [3]. Reflecting a trend to integrate ultrasound early into the care of the critically ill patient, multiple resuscitation protocols have been recently developed [4–26]. Each of these protocols combines many of the same core ultrasound elements, differing mainly in the priority of the exam sequence. 

In this paper, we will discuss two clinical scenarios of hypotension that will highlight how early integration of bedside ultrasound into clinical evaluation can assist in rapid and accurate diagnosis of shock. An easily learned and quickly performed shock ultrasound protocol, the RUSH exam (Rapid Ultrasound in Shock), will be applied in both cases [19, 20]. The RUSH exam involves a 3-part bedside physiologic assessment simplified as “the pump,” “the tank,” and “the pipes.” Several other major resuscitation protocols will be compared to the RUSH exam to describe the core exam elements they share, as well as to demonstrate how they differ.

## 2. Clinical Cases

### 2.1. Case 1

A 72-year-old male presents to the Emergency Department (ED) for evaluation of chest pain, cough, and generalized weakness. He describes the chest pain as sharp and pleuritic, with associated back and upper abdominal pain. His past medical history is significant for hypertension, for which he takes several medications including lisinopril and metoprolol. On physical examination, his vital signs include a blood pressure of 82/60 mm Hg, heart rate 120 beats per minute, respiratory rate 24 breaths per minute, temperature 100.8 F, and pulse oximetry 92% on room air. He is diaphoretic and ill appearing. Lung exam reveals rales in both lung bases, but is otherwise unremarkable. An electrocardiogram (EKG) shows a left bundle branch block, which was present on a test performed one year prior. A portable chest radiograph demonstrates infiltrates at both lung bases, without evidence of a pneumothorax, widened mediastinum, or enlarged cardiac silhouette. 

### 2.2. Case 2

A 64-year-old female with a history of breast cancer presents to the ED with acute shortness of breath and chest pain. She states that the disease has been “stable” and that she has not received chemotherapy in the past three years. She appears acutely ill with blood pressure of 74/58 mm Hg, heart rate 120 beats per minute, respiratory rate 30 breaths per minute, temperature 98 F, and pulse oximetry 94% on room air. Rales are auscultated, but it is difficult to hear heart tones. An EKG reveals a low voltage tracing without ischemic changes. Portable chest radiography demonstrates an enlarged cardiac silhouette and scattered lung opacities.

### 2.3. Case Discussion

In both cases, the patient presents in shock. In the first case, hypotension in a patient with long-standing hypertension indicates significant physiological compromise. While the most likely diagnosis is sepsis due to pneumonia, several clinical questions remain. Could this presentation be the result of a pulmonary embolus, aortic dissection, or myocardial infarction? How much fluid should be given to this patient? His cardiac status is unclear and his heart may not be able to handle a large volume infusion without resultant pulmonary edema. In the second case, a pericardial effusion with tamponade is highly suspected. Should one perform immediate pericardiocentesis? Could this patient be experiencing a massive pulmonary embolus given her history of cancer? Should thrombolysis be considered? Fortunately, an ultrasound machine is available for use in the ED to further evaluate these patients. 

## 3. The RUSH Protocol

### 3.1. Step 1: The Pump

**Figure 1:**
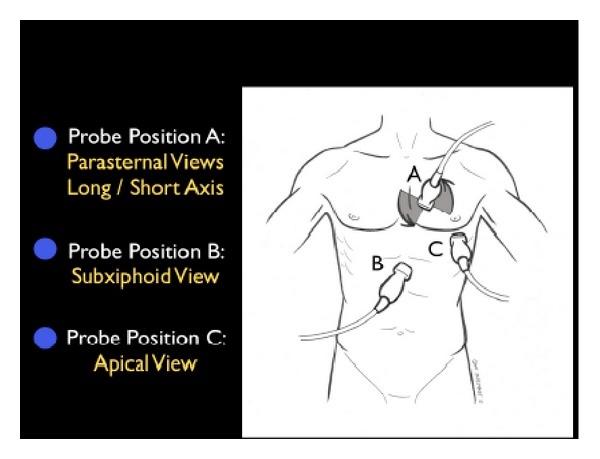
The RUSH exam. Step 1: Evaluation of “the pump”.

The first step in evaluation of the patient in shock is determination of cardiac status, termed for simplicity “the pump.” Imaging of the heart usually involves four classical views: parasternal long and short axis, subxiphoid, and apical (Figure 1). 

 Clinicians caring for the patient in shock should begin with a goal-directed echocardiogram looking for three specific findings: pericardial effusion, left ventricular contractility, and right ventricular dilation. A low-frequency phased array probe is recommended for this exam.

**(A) Pericardial Effusions and Cardiac Tamponade**

**Figure 2:**
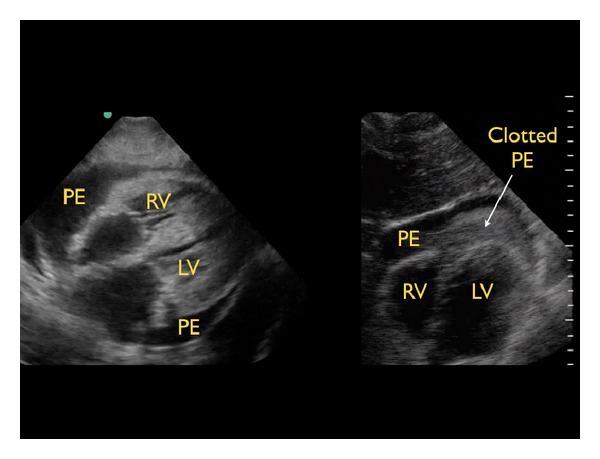
Types of pericardial effusions, subxiphoid cardiac view. Left image: typical effusion, right image: clotted effusion. RV: right ventricle, LV: left ventricle, PE: pericardial effusion.

First, the pericardial sac should be visualized to determine if the patient has a pericardial effusion, which may be the cause of symptoms [27]. Small effusions may be seen as a thin stripe inside the pericardial space, while larger effusions tend to wrap circumferentially around the heart. An exception to this rule may be found in the patient with a loculated effusion, which may exist in both post-operative or post-trauma states and in purulent pericarditis. Fresh fluid or blood tends to have a dark or anechoic appearance, whereas clotted blood or exudates may have a lighter or more echogenic appearance (Figure 2).

**Figure 3:**
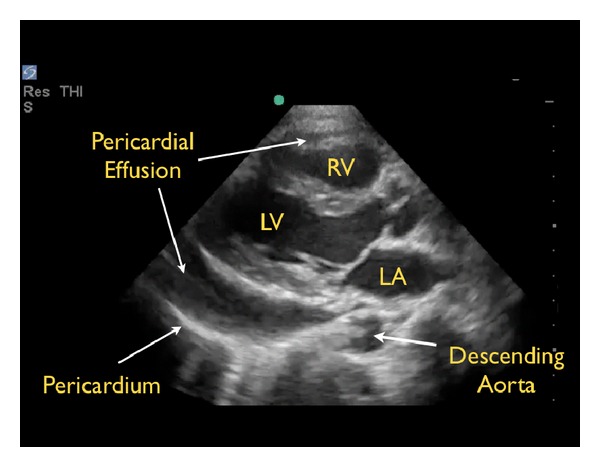
Pericardial effusion, parasternal long axis view. RV: right ventricle, LV: left ventricle, LA: left atrium.

**Figure 4:**
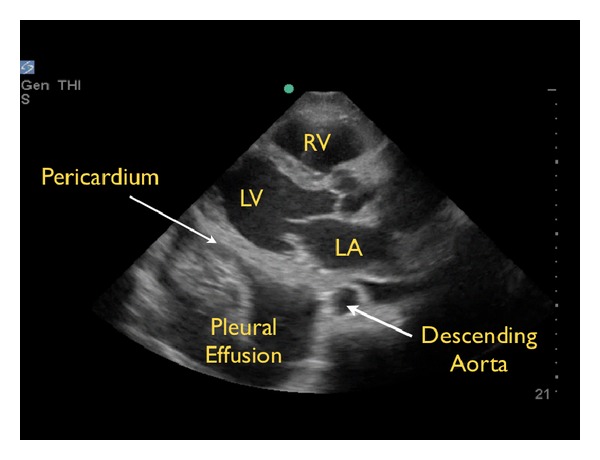
Pleural effusion, parasternal long axis view RV: right ventricle, LV: left ventricle, LA: left atrium.

A pericardial effusion may be confused with a pleural effusion, which is an important distinction. On the parasternal long axis view, a careful evaluation of the fluid in relationship to the descending aorta is critical. Pericardial fluid will be seen anterior to the posterior pericardial reflection and the descending aorta (Figure 3). In contrast, pleural fluid will be seen posterior to the posterior pericardial reflection and the descending aorta (Figure 4). 

**Figure 5:**
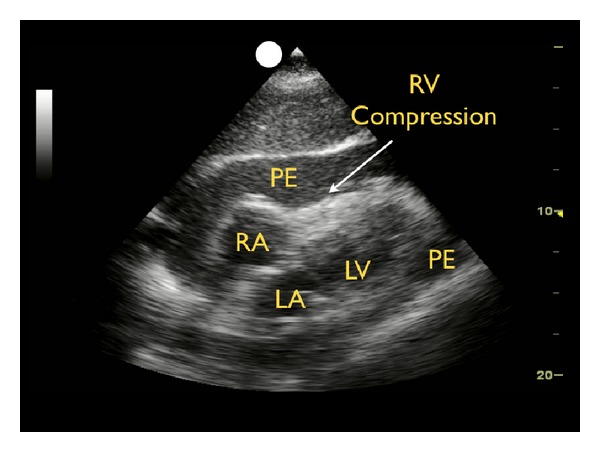
Cardiac tamponade, subxiphoid view. RV: right ventricle, RV: right atrium, LV: left ventricle, LA: left atrium, PE: pericardial effusion.

If a pericardial effusion is identified, the next step is to evaluate the heart for signs of tamponade. Cardiac tamponade results when high pressure within the pericardium prevents the heart from fully expanding and filling during the relaxation phase of the cardiac cycle. Due to the relatively lower pressure in the right side of the heart, evaluation for cardiac tamponade specifically focuses on the movement of the right atrium and ventricle during diastolic filling. Ultrasound findings in tamponade represent a spectrum from subtle inward serpentine deflection of the right atrial and/or the right ventricular wall, to complete diastolic compression of a chamber (Figure 5) [28, 29].

The inferior vena cava (IVC) can also be evaluated for additional confirmatory signs of tamponade; an enlarged plethoric vessel suggests obstructive shock [30]. If tamponade is identified and the patient also displays unstable hemodynamics, an emergent pericardiocentesis is indicated.

**(B) Left Ventricular Contractility**

**Figure 6:**
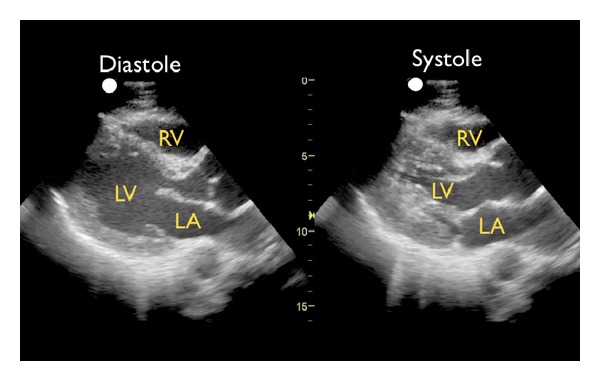
Good left ventricular contractility, parasternal long axis view. RV: right ventricle, LV: left ventricle, LA: left atrium.

**Figure 7:**
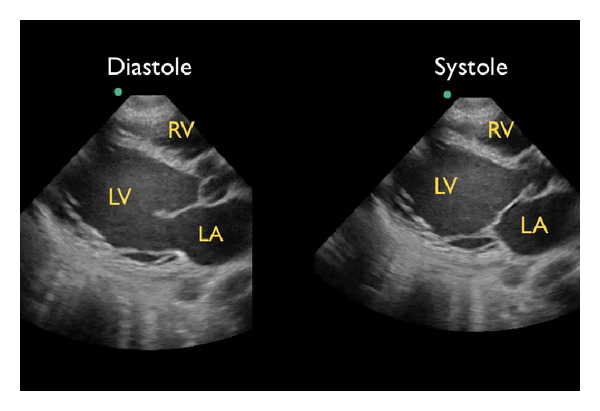
Poor left ventricular contractility, parasternal long axis view. RV: right ventricle, LV: left ventricle, LA: left atrium.

Second, the left ventricle can be analyzed for global contractility. This assessment will give a rapid determination of the strength of “the pump,” which can be critical in guiding fluid resuscitation. The examination focuses on evaluating motion of the left ventricular walls by a visual estimation of the volume change from diastole to systole [31]. A ventricle that has good contractility will have a large volume change between the two cycles (Figure 6). In contrast, a poorly contracting heart will have a small percentage change in the movement of the walls between diastole and systole (Figure 7). The heart may also be dilated in size. Based on these assessments, a patient's contractility can be broadly categorized as being normal, mild-moderately decreased, or severely decreased. A fourth category, known as hyperdynamic, demonstrates small chambers and vigorous, hyperkinetic contractions that may obliterate the ventricle in systole. This is often seen in distributive shock or hypovolemic states.

**Figure 8:**
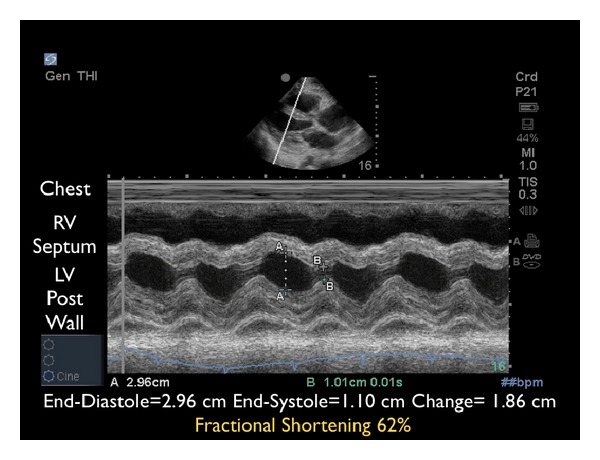
M-mode tracing demonstrating excellent contractility. RV: right ventricle, LV: left ventricle.

**Figure 9:**
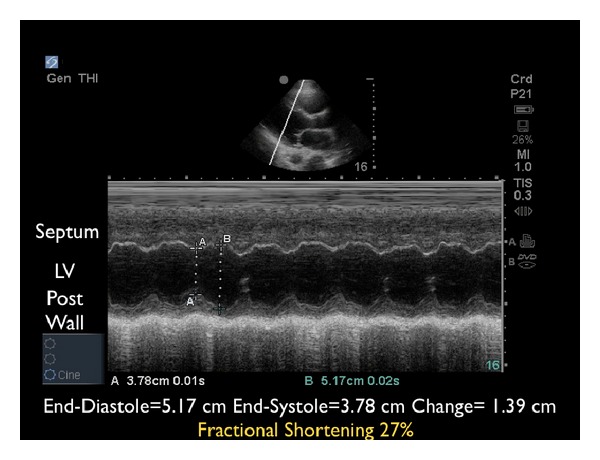
M-mode tracing demonstrating poor contractility. LV: left ventricle.

M-mode can be used to graphically depict the movements of the left ventricular walls through the cardiac cycle. Placing the cursor across the left ventricle just beyond the tips of the mitral valve leaflets, the resultant M-mode tracing allows measurements of the chamber diameter in both systole and diastole. A percentage known as fractional shortening is calculated according to the following formula: [(EDD − ESD)/EDD] × 100, where ESD is end-systolic diameter, measured at the smallest dimension between the ventricular walls, and EDD is the end-diastolic diameter where the distance is greatest. In general, fractional shortening of 30–45% correlates to normal ejection fraction [32]. The M-mode tracing for a hyperdynamic heart shows the left ventricular walls almost touching during systole and a high fractional shortening (Figure 8). In a poorly contracting heart, the M-mode tracing demonstrates wide systolic separation between the ventricular walls and a low fractional shortening (Figure 9). It should be emphasized that fractional shortening does not directly calculate the ejection fraction, rather fractional shortening correlates to overall left ventricular contractility. In comparison to the comprehensive and relatively time intensive volumetric assessments for measuring ejection fraction, fractional shortening is a semiquantitative method for determining systolic function that is fast and easy to perform and can provide critical data to guide resuscitation [33].

**Figure 10:**
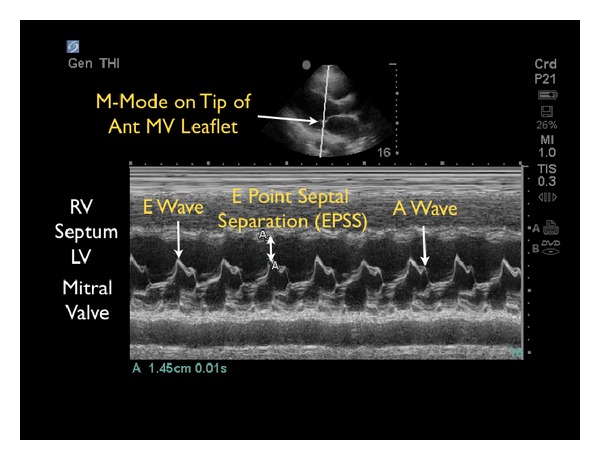
E-point septal separation with decreased contractility. M-mode Doppler tracing. RV: right ventricle, LV: left ventricle.

Motion of the anterior leaflet of the mitral valve can also be used to assess contractility. In a normal contractile state, the anterior mitral leaflet can be seen in the parasternal long-axis view touching or closely approaching the septal endocardium in early diastole. The degree of excursion of the mitral valve directly correlates with the contractile state of the left ventricle. As cardiac contractility decreases, the distance between the mitral valve and septum increases. M-mode is used to document and measure the degree of mitral valve excursion, known as the E-point septal separation (EPSS) (Figure 10). To obtain this measurement, the M-mode cursor is placed over the tip of the anterior mitral leaflet. As the mitral valve moves during diastole, the M-mode tracing reveals a characteristic pattern of two repeating waves. The first is the E-wave, which reflects the initial and maximal opening of the valve to allow passive filling of the left ventricle. Immediately following is the A-wave, which is usually smaller and corresponds to left atrial contraction. The EPSS is the minimal distance between the E-wave and the septum and is normally less than 7 mm [34]. Studies have demonstrated that EPSS greater than 1 cm reliably correlates with a low ejection fraction [35]. Another study demonstrated that Emergency Physicians are able to accurately estimate ejection fraction using EPSS [36], highlighting its value in identifying patients with abnormal contractility. An important caveat is that EPSS does not reflect systolic dysfunction in the setting of mitral valve abnormalities (stenosis, regurgitation), aortic regurgitation, or extreme left ventricle hypertrophy.

**(C) Right Ventricular Size**

**Figure 11:**
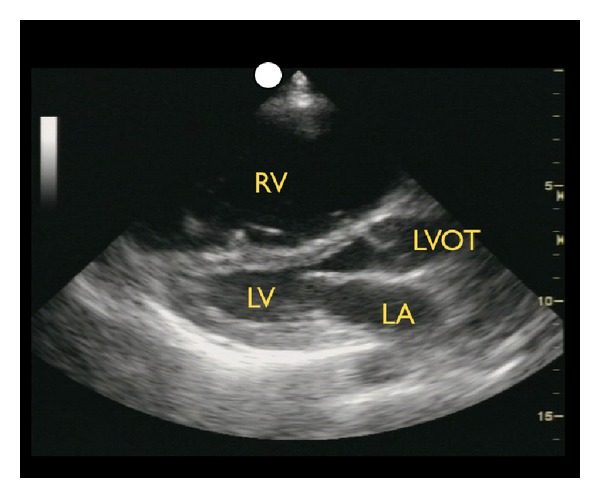
Right ventricular dilation, parasternal long axis view. RV: right ventricle, LA: left atrium, LV: left ventricle, LVOT: left ventricle outflow tract.

The third goal-directed examination of the heart focuses on the evaluation of right heart strain, a potential sign of a large pulmonary embolus in the patient in shock. Any condition that causes a sudden pressure increase within the pulmonary vascular circuit will result in acute dilation of the right side of the heart. On bedside echocardiography, the normal ratio of the left-to-right ventricle is 1 : 0.6. Dilation of the right ventricle, especially to a size greater than the left ventricle, may be a sign of a large pulmonary embolus in the hypotensive patient (Figure 11) [37, 38]. In addition, deflection of the inter-ventricular septum toward the left ventricle signals higher pressures within the right side of the heart and the pulmonary artery [39, 40]. In this situation, the exam should proceed directly to evaluation of the leg veins for a deep vein thrombosis (DVT). When the right ventricular wall is also thickened, right ventricular dilation may be more indicative of a chronic illness such as long-standing pulmonary hypertension.

### 3.2. Step 2: The Tank

**Figure 12:**
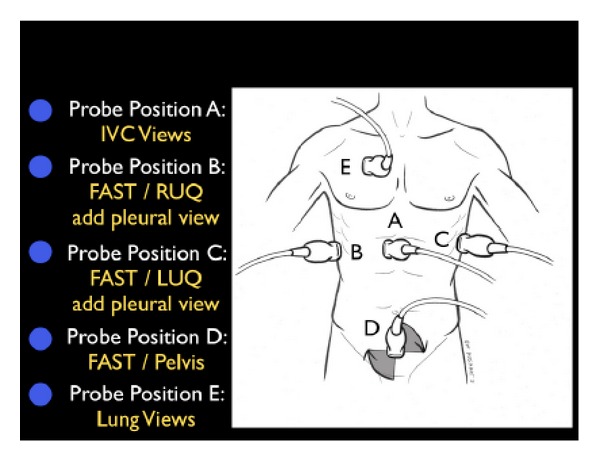
The RUSH exam. Step 2: Evaluation of “the tank”.

The second part of the RUSH protocol focuses on the determination of the effective intravascular volume status, which will be referred to as “the tank” (Figure 12).

**(A) Fullness of the Tank: Inferior Vena Cava and Internal Jugular Veins**

The first step assesses “fullness of the tank” by examining the IVC. Looking at both the relative vessel size and its respiratory dynamics, the IVC provides an indication of intravascular volume and has been used to estimate the central venous pressure (CVP) [41–46]. To image the IVC, the probe is placed in the standard 4-chamber subxiphoid position to first identify the right ventricle and the right atrium. The probe is then rotated posteriorly toward the spine with the marker laterally oriented, examining for the convergence of the IVC with the right atrium. The IVC should be followed inferiorly as it passes through the liver, specifically looking for the confluence of the three hepatic veins with the IVC. The IVC diameter should be evaluated just inferior to this point, at a position approximately 2 cm from the junction of the right atrium and the IVC [47]. The IVC can also be imaged in the long-axis plane. The IVC will be found directly to the right of the aorta and can be differentiated by its thinner walls and respiratory flow variation on Color Doppler imaging. As the patient breathes, the IVC will have a normal pattern of collapse during inspiration. This is due to the negative pressure generated in the chest with inspiration, leading to increased blood flow from the abdominal to the thoracic cavity. This respiratory variation can be further augmented by having the patient sniff, or inspire, forcefully. This combination of IVC size and the percentage change during inspiration, termed sonospirometry, has been shown to accurately estimate the CVP [48, 49]. M-mode sonography of the IVC provides an excellent means to measure and document the degree of inspiratory IVC collapse.

**Figure 13:**
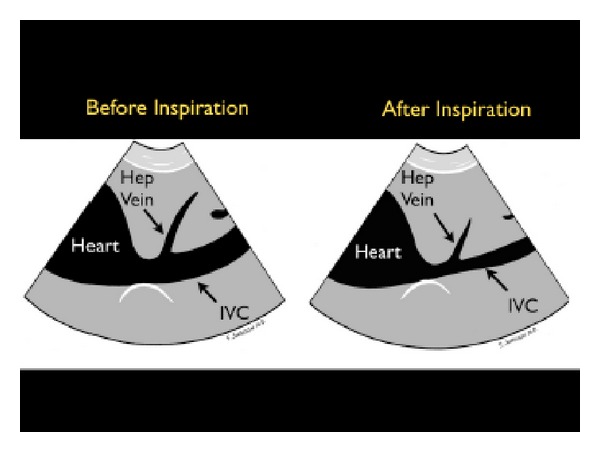
Collapsible inferior vena cava, long axis view.

**Figure 14:**
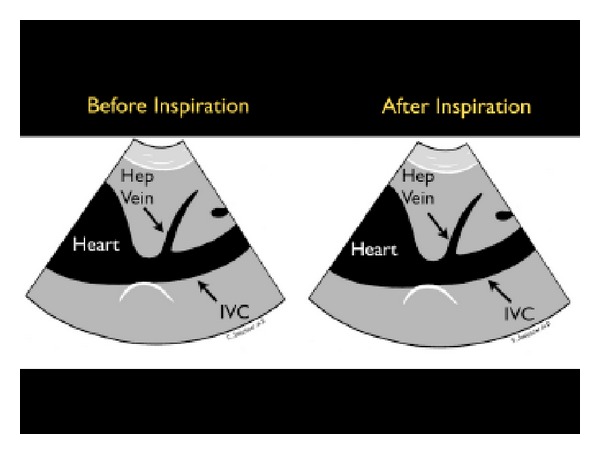
Inferior vena cava plethora, long axis view.

Newly published guidelines by the American Society of Echocardiography support the general use of evaluation of IVC size and collapsibility in assessment of CVP [50]. The recommendations are that an IVC diameter <2.1 cm that collapses >50% with sniff correlates to a normal CVP pressure of 3 mm Hg (range 0–5 mm Hg). This phenomenon may be observed in hypovolemic and distributive shock states (Figure 13). A larger IVC >2.1 cm that collapses <50% with sniff suggests a high CVP pressure of 15 mm Hg (range 10–20 mm Hg). This phenomenon may be seen in cardiogenic and obstructive shock states (Figure 14). In scenarios in which the IVC diameter and collapse do not fit this paradigm, an intermediate value of 8 mm Hg (range 5–10 mm) is suggested. Note that in the intubated patient receiving positive pressure ventilation, the respiratory dynamics of the IVC will be reversed. However, in most intubated patients, the IVC becomes larger and less compliant [51]. Therefore, repeated examinations of the IVC with fluid loading may be more helpful than a static one-time measurement, as volume responsiveness of the patient has been correlated with progressive filling of the IVC over time.

The current prevailing opinion recommends that assessment of cardiac function be performed prior to measurement of the IVC to provide context for the interpretation of IVC findings. Examination of the IVC in both short and long-axis views is also emphasized, as a single longitudinal measurement may be off-axis, incorrectly underestimating the size of the vessel in a pitfall known as the cylinder tangent effect.

**Figure 15:**
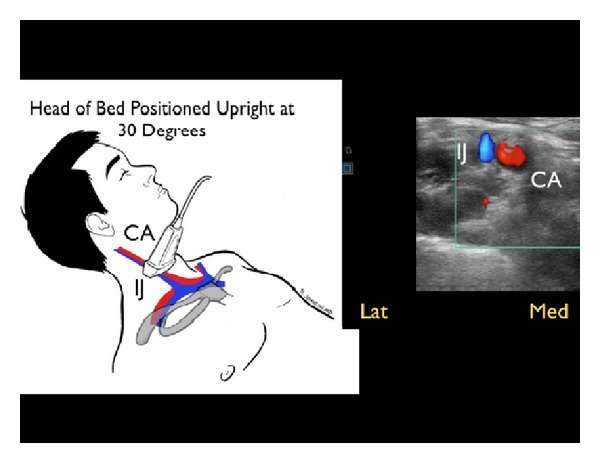
Small, collapsing internal jugular vein, short axis view. IJ: internal jugular vein, CA: carotid artery.

**Figure 16:**
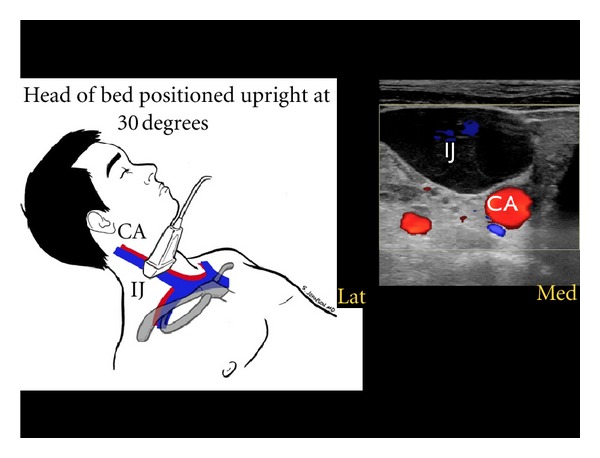
Large, distended internal jugular vein, short axis view. IJ: internal jugular vein, CA: carotid artery.

In the patient in whom a gas filled stomach or intestine precludes adequate assessment of the IVC, the internal jugular (IJ) veins may be evaluated with the head of the patient's bed elevated to 30 degrees. A high-frequency linear array probe is recommended for this exam. For volume assessment, one should examine both the relative fullness of the veins and the height of the vessel column in the neck, as well as the percentage change in these parameters with respiratory dynamics [52, 53]. Optimally, both right and left IJ veins should be evaluated. A small caliber jugular vein, with a closing level low in the neck with inspiration, correlates well with a low CVP (Figure 15). Conversely, an IJ vein that is distended superiorly to the angle of the jaw, with little inspiratory collapse, indicates an elevated CVP (Figure 16). This provides a sonographic evaluation for JVD and can be helpful in corroborating the volume assessment made from evaluation of the IVC [54].

**(B) Leakiness of the Tank: FAST and Thoracic Ultrasound**

**Figure 17:**
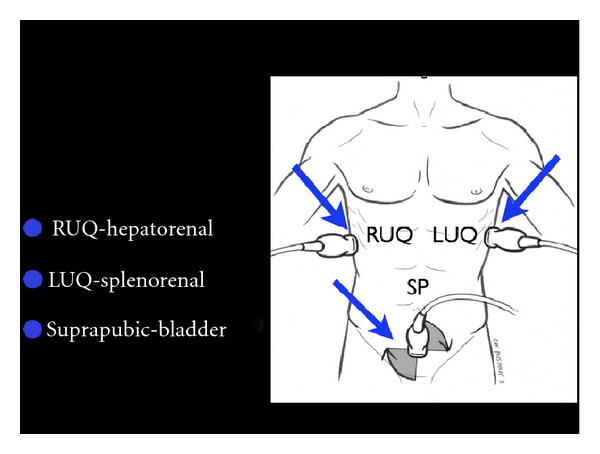
FAST exam.

**Figure 18:**
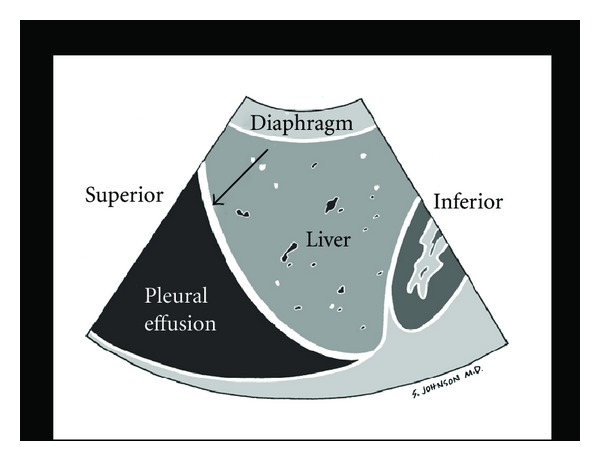
Pleural effusion.

Once a patient's intravascular volume status has been determined, the next step is to look for “leakiness of the tank.” This refers to hemodynamic compromise due to a loss of critical fluids from the core vascular circuit. In traumatic conditions, the clinician must quickly determine whether hemoperitoneum or hemothorax is present. In this setting, hypovolemic shock occurs as a result of “a hole in the tank.” In nontraumatic conditions, accumulation of excess fluid into the abdominal and chest cavities often signifies “tank overload,” with resultant pleural effusions and ascites that may build up with failure of the heart, kidneys, and/or liver. In a female patient of childbearing age, the exam should specifically assess for free abdominal or pelvic fluid, findings which may indicate a ruptured ectopic pregnancy. This assessment is initiated with the Focused Assessment of Sonography for Trauma (FAST) exam (Figure 17) [55]. Aiming the probe above the diaphragm will allow for identification of a thoracic fluid collection (Figure 18). If an abnormal fluid collection is detected and there is a suspicion of a corresponding infectious process, ultrasound-guided aspiration of the fluid can be performed.

**Figure 19:**
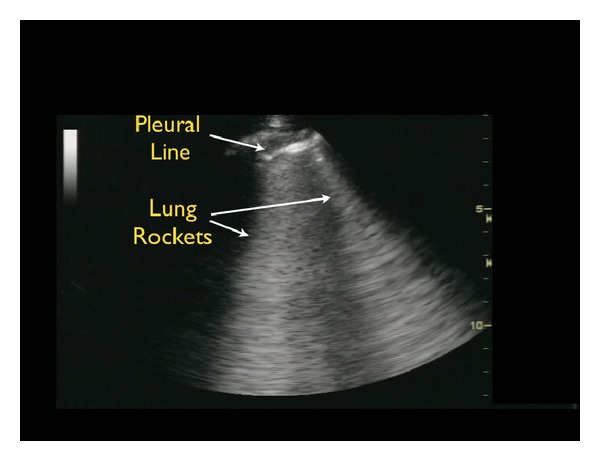
Pulmonary edema B-lines.

Finally, lung ultrasound can identify pulmonary edema, a sign often indicative of “tank overload” and “tank leakiness” with fluid accumulation in the lung parenchyma [56–59]. To assess for pulmonary edema with ultrasound, the lungs are scanned with a low-frequency phased array transducer in the anterolateral chest between the second and fifth rib interspaces. Examining the lungs from a more lateral orientation, or even from a posterior approach, increases the sensitivity of this technique [60]. Detection of pulmonary edema with ultrasound relies on seeing a special type of lung ultrasound artifact, termed ultrasound B-lines or “lung rockets.” These B-lines appear as a series of diffuse, brightly echogenic lines originating from the pleural line and projecting posteriorly in a fanlike pattern (Figure 19). In contrast to the smaller comet tail artifacts of normal lung that fade out within a few centimeters of the pleura and are better seen with the use of a high frequency probe, the B-lines of pulmonary edema are more defined and extend to the far field of the ultrasound image with use of a low-frequency probe.

**(C) Compromise of the Tank: Pneumothorax**

**Figure 20:**
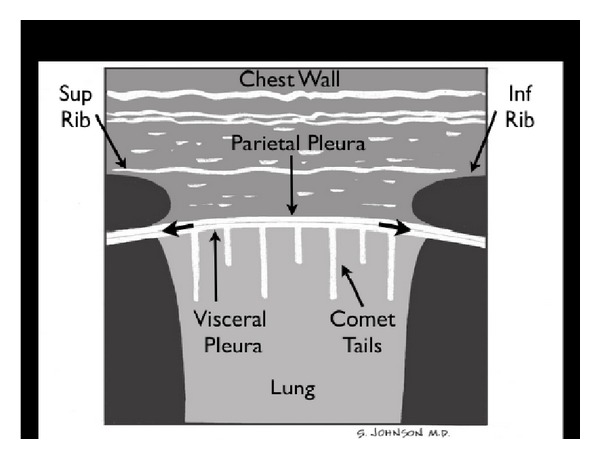
Normal lung.

**Figure 21:**
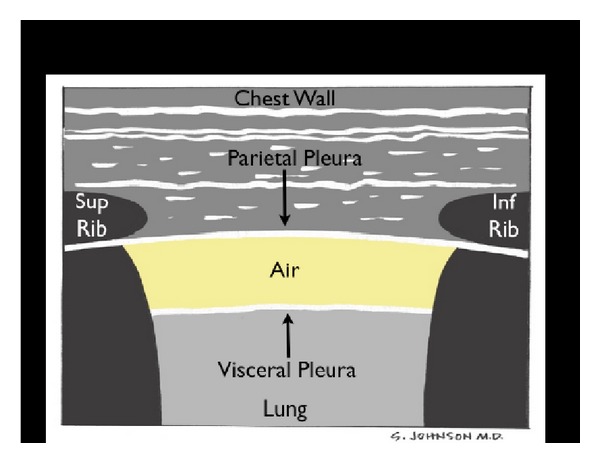
Pneumothorax.

The third component of the assessment of the tank is to assess for “tank compromise.” This may occur as a result of a tension pneumothorax, where venous return to the heart is limited by increased thoracic pressure. For this exam, the patient should be positioned in a supine position. A high frequency linear array probe is positioned at the most anterior point of the thorax to identify the pleural line. This line appears as an echogenic horizontal line, located approximately a half-centimeter deep to the ribs. The pleural line consists of the closely opposed visceral and parietal pleura. In the normal lung, the visceral and parietal pleura can be seen to slide against each other, with a glistening or shimmering appearance as the patient breathes. “Comet-tail” artifacts, or vertical hyperechoic lines, may be seen to extend posteriorly from the opposed pleura (Figure 20). The presence of lung sliding with comet-tails excludes a pneumothorax. In contrast, a pneumothorax results in air collecting between the layers of the parietal and visceral pleura, preventing the ultrasound beam from detecting normal lung sliding and vertical comet-tails (Figure 21) [61–64]. The pleural line will consist only of the parietal layer, seen as a single stationary line. While the line may be seen to move anteriorly and posteriorly due to exaggerated chest wall motions, noted often in cases of severe respiratory distress, the characteristic horizontal sliding of the pleural line will not be seen.

**Figure 22:**
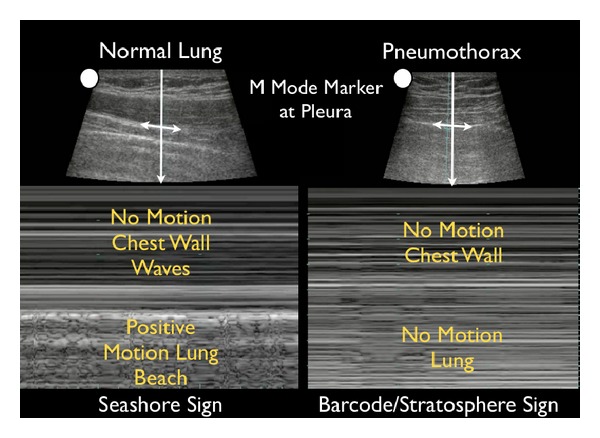
M-mode of normal lung versus pneumothorax.

The presence or absence of lung sliding can be graphically depicted using M-mode Doppler. A normal image will depict “waves on the beach.” Closest to the probe, the stationary anterior chest wall demonstrates a linear pattern, while posterior to the pleural line the presence of lung motion demonstrates an irregular, granular pattern. In pneumothorax, M-mode Doppler ultrasound will only show repeating horizontal lines, indicating a lack of lung sliding in a finding known as the “barcode” or “stratosphere sign” (Figure 22). 

### 3.3. Step 3: The Pipes

**Figure 23:**
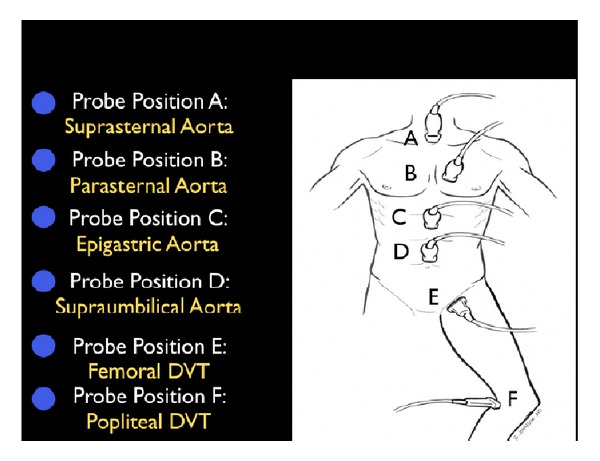
The RUSH exam. Step 3: Evaluation of “the pipes”.

The third and final step in the RUSH exam is to examine “the pipes,” looking first at the arterial side of the circulatory system, and secondly, at the venous side (Figure 23). Vascular catastrophes, such as a ruptured abdominal aortic aneurysm (AAA) or an aortic dissection, are life-threatening causes of hypotension. The survival of such patients may often be measured in minutes, and the ability to quickly diagnose these diseases is crucial.

**(A) Rupture of the Pipes: Aortic Aneurysm and Dissection**

**Figure 24:**
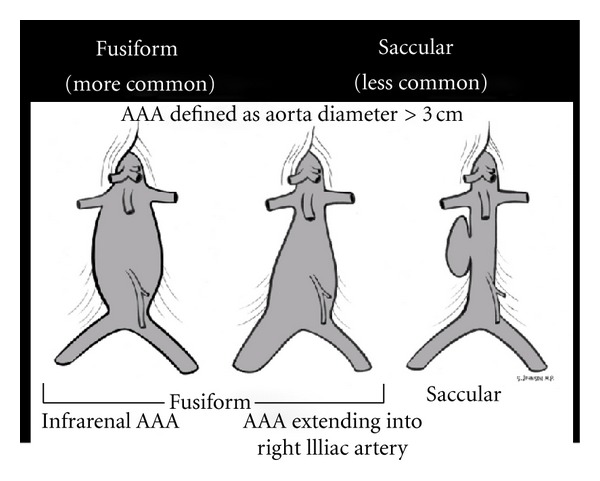
Abdominal aortic aneurysm (AAA) types.

**Figure 25:**
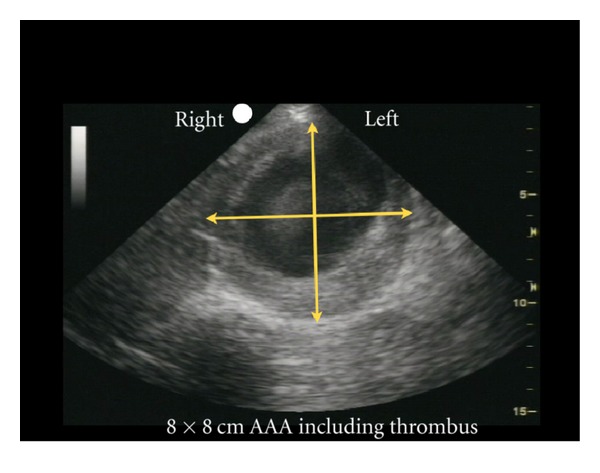
Abdominal aortic aneurysm (AAA) measured, short axis view.

Examination of the abdominal aorta along its entire course is essential to rule out an aneurysm, paying special attention to the aorta below the renal arteries where most AAAs are located (Figure 24). An AAA is diagnosed when the vessel diameter exceeds 3 cm. Measurements should be obtained in the short-axis plane, measuring the maximal diameter of the aorta from outer wall to outer wall and should include any thrombus present in the vessel (Figure 25). Smaller aneurysms may be symptomatic, although rupture is more common with aneurysms measuring larger than 5 cm [65–67]. Rupture of an AAA typically occurs into the retroperitoneal space, which is an area difficult to visualize with ultrasound. Therefore, in an unstable patient with clinical symptoms consistent of this condition and an AAA diagnosed by ultrasound, rupture should be assumed and emergency treatment expedited.

**Figure 26:**
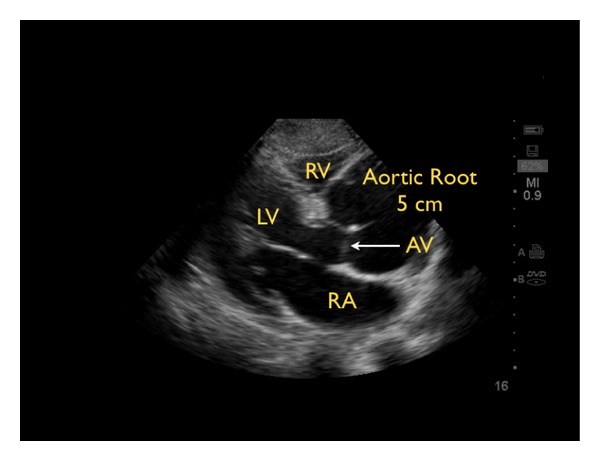
Aortic arch dissection with widened aortic root, parasternal long axis view. RV: right ventricle, LV: left ventricle, LA: left atrium, AV: aortic valve.

Another crucial part of “the pipes” protocol is evaluation for an aortic dissection. Sonographic findings suggestive of the diagnosis include the presence of aortic root dilation and an aortic intimal flap [68–70]. The parasternal long-axis view of the heart permits an evaluation of the proximal aortic root. In general, a normal aortic root should measure less than 3.8 cm. A Stanford Class A aortic dissection will often lead to a widened aortic root (Figure 26) [71]. In this type of dissection, aortic regurgitation or pericardial effusion may also be seen. An echogenic intimal flap may be recognized within the dilated root or along the course of the aorta. The suprasternal view allows further imaging of the aortic arch. A Stanford Class B aortic dissection may be detected by noting the presence of an intimal flap in the descending aorta or in the abdominal aorta in cases that propagate below the diaphragm. Color flow Doppler imaging can be used to confirm this diagnosis, by further delineating two lumens with distinct blood flow within the vessel. Immediate Cardiothoracic Surgical consultation should be obtained with suspicion of an ascending aortic dissection in an unstable patient. This is especially important in the patient with an aortic dissection resulting in a pericardial effusion, since the optimal therapy is surgical. Emergent pericardiocentesis in this setting has precipitated worse outcomes in patients through the massive reaccumulation of pericardial blood [72].

**(B) Obstruction of the Pipes: DVT**

**Figure 27:**
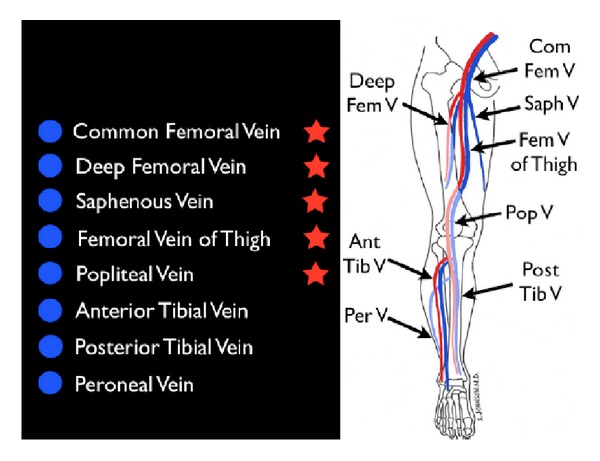
Limited leg deep venous thrombosis exam (starred veins).

If a thromboembolic event is suspected as the cause of shock, the next step should be an assessment of the venous side of “the pipes.” Since the majority of pulmonary emboli originate from lower extremity DVTs, the examination is concentrated on a limited compression evaluation of specific areas of the leg (Figure 27). Simple compression ultrasonography, which uses a high frequency linear probe to apply direct pressure to the vein, has good overall sensitivity for detection of DVT [73, 74].

**Figure 28:**
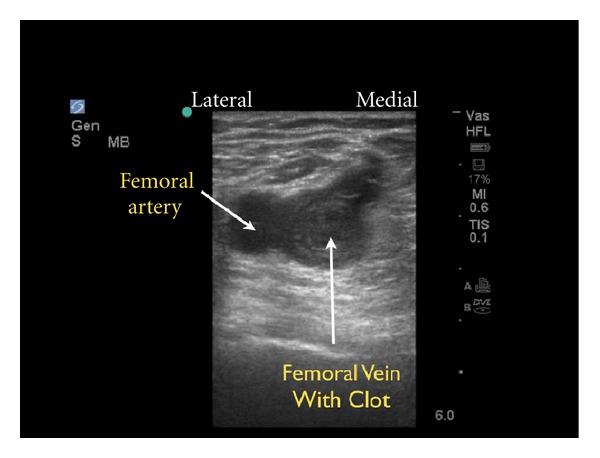
Deep venous thrombosis of the femoral vein, short axis view.

A normal vein will completely collapse with simple compression. In contrast, an acute blood clot will form a mass within the lumen of the vein. The pathognomonic finding of DVT is the incomplete compression of the anterior and posterior walls of the vein with applied probe pressure (Figure 28). The limited DVT examination has been found to have a high accuracy for evaluation of clot within the leg veins and can be rapidly performed. The exam focuses on the common femoral vein, the proximal femoral vein of the thigh and the popliteal vein behind the knee [75–78]. If an upper extremity thrombus is clinically suspected, the same compression techniques can be employed on the arm veins.

## 4. Putting RUSH into Action

The mnemonic of the RUSH protocol—pump, tank, and pipes—was created as a physiological roadmap for clinicians to easily remember in the heat of resuscitation.  Table 1 summarizes the components of the RUSH exam. The RUSH protocol was designed to be rapidly performed by choosing those specific exam components that are most applicable to the clinical context. While the entire protocol is extensive and incorporates multiple ultrasound elements, it is advised that clinicians always start with evaluation of the heart and IVC/IJ veins. The RUSH exam should then be tailored based on clinical suspicion, as many patients may be assessed with an abbreviated exam. Incorporation of other components, such as lung, FAST, aorta, and DVT exams can be determined as the clinical picture dictates.  Table 2 demonstrates how using the RUSH exam at the bedside can assist in the diagnosis of shock in the critically ill patient.

## 5. Overview of Current Resuscitation ****Ultrasound Protocols

As bedside ultrasound is increasingly available and incorporated into Critical Care and Emergency Medicine, a number of protocols have been developed for the evaluation of patients in shock, respiratory distress, and cardiac arrest. Major resuscitation ultrasound protocols for use in critically ill medical and trauma patients include: ACES [4], BEAT [5], BLEEP [6], Boyd Echo [7], EGLS [8], Elmer/Noble Protocol [9], FALLS [10], FAST [11], Extended-FAST [12], FATE [13], FEEL-Resuscitation [14], FEER [15], FREE [16], POCUS-Fast and Reliable [17], RUSH-HIMAP [18], RUSH-Pump/Tank/Pipes [19, 20], Trinity [21] and UHP [22]. See Table 3 for comparison of the major medical shock ultrasound protocols. Current major resuscitation protocols for dyspnea include the BLUE protocol [23] and RADIUS [24]. The BLUE protocol focuses solely on lung ultrasound for the diagnosis of the following conditions: pneumothorax, pulmonary edema, pulmonary consolidation, and effusions. The RADIUS protocol begins with cardiac and IVC evaluation, followed by a focused pulmonary exam.

While it appears that there are many competing protocols, what unifies these protocols is an emphasis on many of the same ultrasound examination components. Cardiac and IVC views are integral to the majority. The cardiac evaluation in these protocols emphasizes the following: focused evaluation for pericardial effusion and tamponade, left ventricular contractility, and right ventricular strain. Cardiac valvular assessment remains absent from most Emergency Medicine protocols, although mentioned in some Critical Care exams. More recent protocols have included lung ultrasound as an important component. While these protocols may prioritize the sequence of these components differently, the summary conclusion is that these many shock ultrasound guidelines hold more in common than in difference.

## 6. Conclusion of Patient Cases

In the first clinical case, the patient was clinically diagnosed with septic shock. The Rush exam was performed and the first step, evaluation of “the pump” demonstrated no pericardial effusion, good cardiac contractility and no evidence of right ventricular strain. Evaluation of “the tank” demonstrated relative hypovolemia and a low CVP, with a small diameter IVC that collapsed completely with sniffing. A small diameter and collapsible IJ vein evaluation corroborated this volume assessment. Evaluation of “the pipes” demonstrated a normal thoracic and abdominal aorta size. The DVT exam was deferred given the low probability of DVT from the clinical context. Based on this hemodynamic evaluation, the patient was aggressively resuscitated with normal saline, and periodic ultrasound reevaluation demonstrated IVC filling with volume loading. His blood pressure improved with this resuscitation and broad-spectrum antibiotics were administered. Blood testing for troponin elevation over time was negative and further advanced imaging of his aorta while admitted to the hospital was normal.

In the second case, the RUSH exam immediately detected a large pericardial effusion on evaluation of “the pump”. Diastolic compression of the right ventricle was noted, indicating tamponade physiology. Rapid assessment of “the tank” demonstrated a large diameter IVC that had little respiratory change, confirming an elevated CVP and corroborating the presence of tamponade physiology. An ultrasound-guided pericardiocentesis was performed within minutes of the patient's presentation to the ED. Following the procedure, the patient's hemodynamics improved, and she was admitted to the intensive care unit. 

These cases highlight the role of resuscitative ultrasound and the RUSH protocol in guiding the care of the patient in shock. Due to the noninvasive nature of ultrasound and its ability to provide repeated assessment of physiology during resuscitation, this modality has moved to the front line of emergency care and is considered among the new and important uses of ultrasound by the American College of Emergency Physicians and Critical Care Societies [79–82]. Physicians caring for critical patients should strongly consider integrating focused ultrasound techniques, such as the RUSH exam, into their resuscitation pathways to augment their clinical evaluation and guide resuscitation.

## Figures and Tables

**Table 1:** RUSH protocol summary.

RUSH exam	Hypovolemic shock	Cardiogenic shock	Obstructive shock	Distributive shock
Pump	Hypercontractile heart Small heart size	Hypocontractile heart Dilated heart size	Pericardial effusion, RV strain Hypercontractile heart	Hypercontractile heart (early sepsis) Hypocontractile heart (late sepsis)

Tank	Flat IVC Flat IJV Peritoneal fluid Pleural fluid	Distended IVC Distended IJV Lung rockets Pleural effusions, ascites	Distended IVC Distended IJV Absent lung sliding (PTX)	Normal/small IVC Normal/small IJV Pleural fluid (empyema) Peritoneal fluid (peritonitis)

Pipes	AAA Aortic dissection	Normal	DVT	Normal

**Table 2:** Using the RUSH protocol to diagnose the type of shock.

	Step no. 1	Step no. 2	Step no. 3
Pump	Pericardial effusion: (a) Effusion present? (b) Signs of tamponade? Diastolic collapse of R Vent +/− R Atrium?	Left ventricular contractility: (a) Hyperdynamic? (b) Normal? (c) Decreased?	Right ventricular strain: (a) Increased size of RV? (b) Septal displacement from right to left?

Tank	Tank volume: (1) Inferior vena cava: (a) Large size/small Insp collapse? —CVP high— (b) Small size/large Insp collapse? —CVP Low— (2) Internal jugular veins: (a) Small or large?	Tank leakiness: (1) E-FAST exam: (a) Free fluid Abd/Pelvis? (b) Free fluid thoracic cavity? (2) Pulm edema: Lung rockets?	Tank compromise: Tension pneumothorax? (a) Absent lung sliding? (b) Absent comet tails?

Pipes	Abdominal aorta aneurysm: Abd aorta > 3 cm?	Thoracic aorta aneurysm/dissection: (a) Aortic root > 3.8 cm? (b) Intimal flap? (c) Thor aorta > 5 cm?	(1) Femoral vein DVT? Noncompressible vessel? (2) Popliteal vein DVT? Noncompressible vessel?

**Table 3:** Summary of the major ultrasound protocols for medical shock assessment.

Protocol	ACES [4]	BEAT [5]	BLEEP [6]	Boyd: ECHO [7]	EGLS [8]	Elmer/Noble [9]	FALLS [10]	FATE [13]	FEEL: RESUS [14]	FEER [15]	FREE [16]	POCUS [17]	RUSH: HIMAP [18]	RUSH: Pump Tank Pipes [19, 20]	Trinity [21]	UHP [22]
Cardiac	1	1	1	1	2	1	3	1	1	1	1	3	1	1	1	3
IVC	2	2	2	2	3	2	4					4	2	2		
FAST A/P	4					3						1	3	3	3	1
Aorta	3											5	4	7	2	2
Lungs PTX					1	4	2					2	5	6		
Lungs effusion	5							2						4		
Lungs edema					4	5	1					6		5		
DVT												7		8		
Ectopic Pregnancy												8				

Numbers indicate exam sequence for each protocol.
